# Bispectral index-guided versus standard anaesthesia monitoring for intraoperative awareness prevention

**DOI:** 10.6026/973206300220931

**Published:** 2026-02-28

**Authors:** Brajesh Kumar, Nancy Ekka, Aditya Singh Chauhan, Sourabh Shrivastava

**Affiliations:** 1 Consultant Anaesthesiologist, Bansal Hospital, Sagar, Madhya Pradesh, India; 2 Department of Anaesthesiology, Gajra Raja Medical college Gwalior, Madhya Pradesh, India

**Keywords:** Bispectral index (BIS), intraoperative awareness, depth of anaesthesia monitoring, sevoflurane consumption, recovery profile, haemodynamic stability, explicit recall

## Abstract

Although bispectral index (BIS) monitoring provides an objective measure of hypnotic depth, its routine use to prevent awareness remains debated due to conflicting evidence and cost considerations. Therefore, it is of interest to evaluate BIS-guided versus clinically guided sevoflurane anaesthesia in 200 adults undergoing elective surgery over 60 minutes. No definite intraoperative awareness occurred in either group, confirmed by modified Brice interviews up to 30 days postoperatively. BIS monitoring reduced sevoflurane consumption by 24% (1.7 ± 0.4 vs 2.2 ± 0.5 MAC-hours; p < 0.001) and accelerated extubation (7.9 ± 2.3 vs 10.8 ± 3.1 min; p < 0.001) and PACU discharge (46.2 ± 11.8 vs 56.9 ± 14.7 min; p < 0.001). It also provided greater haemodynamic stability, with lower MAP variability (11.4 ± 3.9% vs 16.8 ± 5.6%; p < 0.001). BIS monitoring optimizes anaesthetic delivery and recovery efficiency without lowering awareness incidence compared to vigilant clinical monitoring.

## Background:

Intraoperative awareness with explicit recall is a rare but devastating complication of general anaesthesia that can lead to post-traumatic stress disorder, chronic anxiety and lasting psychological harm [1]. Large-scale audits have reported an overall incidence of 1:19,000 in low-risk patients but up to 1:670 in high-risk groups such as cardiac, trauma, or obstetric surgery using neuromuscular blockade [2, 3]. Traditional assessment of anaesthetic depth relies on surrogate clinical signs including heart rate, blood pressure, sweating, lacrimation and movement. These parameters are unreliable, particularly when β-blockers, vasodilators, or muscle relaxants are used and they correlate poorly with consciousness [4]. Consequently, processed electroencephalographic monitors such as the bispectral index (BIS) were developed to provide a direct, objective measure of hypnotic effect [5]. BIS integrates power spectrum, bispectral analysis and burst suppression ratio into a single dimensionless value (0-100), with 40-60 generally accepted as the target range for surgical anaesthesia [6]. Early enthusiasm followed the B-Aware trial, which showed an 82% reduction in awareness among high-risk patients when BIS was used compared with routine care [7]. However, subsequent large trials yielded conflicting results. The B-Unaware trial found no difference between BIS and end-tidal anaesthetic concentration (ETAC) alerting [8]. And the BAG-RECALL trial similarly reported no benefit of BIS over ETAC protocols in preventing awareness [9]. A Cochrane review concluded that BIS reduces awareness only in specific high-risk contexts but offers consistent benefits in anaesthetic drug reduction and faster recovery [10]. Despite extensive investigation, controversy persists regarding the routine application of BIS monitoring, particularly in moderate-risk surgical populations where awareness incidence is already very low with modern anaesthetic techniques. Few studies have directly compared BIS-guided titration against contemporary clinical practice without an ETAC alarm protocol in a blinded fashion. Therefore, it is of interest to evaluate whether BIS monitoring confers additional protection against awareness while quantifying its impact on anaesthetic efficiency, recovery profile and intraoperative stability in a general surgical population.

## Materials and Methods:

This prospective, randomized, parallel-group, assessor-blinded clinical trial was conducted between April 2022 and March 2023. Sample size calculation was based on an expected awareness incidence of 0.2% in the control group and aimed to detect a 90% relative reduction (to 0.02%) with 90% power and α = 0.05. This required approximately 45,000 patients per group, which was clearly impractical. Therefore, following contemporary trials, we powered the study to detect a 20% reduction in sevoflurane consumption (primary secondary endpoint), with SD 0.5 MAC-hours, yielding 96 patients per group. Two hundred patients were enrolled to account for dropouts and protocol violations. Adults aged 18-70 years, ASA physical status I-III, scheduled for elective non-cardiac, non-neurosurgical procedures expected to last >60 minutes under general anaesthesia with tracheal intubation and neuromuscular blockade were eligible. Exclusion criteria included known neurological or psychiatric disease, chronic opioid or benzodiazepine use, alcohol or substance abuse, body weight <40 or >120 kg, anticipated difficult airway, emergency surgery and procedures precluding BIS sensor placement (e.g., forehead surgery). Randomization (1:1) was performed using computer-generated blocks of 10 with allocation concealment via sequentially numbered opaque sealed envelopes. Patients were assigned to BIS-guided anaesthesia (Group B) or standard clinical practice (Group C). Anaesthetists were not blinded due to the visible BIS display, but postoperative interviewers and recovery nurses remained blinded to group allocation. All patients received premedication with oral alprazolam 0.25 mg the night before and 2 hours preoperatively. Standard monitoring included ECG, non-invasive blood pressure, spo_2_, etco_2_, neuromuscular transmission (TOF-Watch SX) and temperature. Anaesthesia was induced with fentanyl 2 µg/kg, propofol 2-2.5 mg/kg and rocuronium 0.8 mg/kg. Maintenance was with sevoflurane in oxygen-air (fio_2_ 0.4-0.5). In Group B, the BIS Vista monitor (Medtronic, USA) sensor was applied before induction and sevoflurane concentration was titrated to maintain BIS 40-60 throughout surgery. In Group C, the BIS display was covered and sevoflurane was adjusted according to standard clinical signs (heart rate, blood pressure ±20% of baseline, lacrimation, sweating and movement). Additional propofol boluses (20-50 mg) were permitted in both groups for suspected light anaesthesia. Hypotension (MAP <60 mmhg) was treated with fluids or ephedrine; hypertension or tachycardia with additional fentanyl or esmolol as required. At skin closure, sevoflurane was discontinued and neuromuscular blockade was reversed with sugammadex 2-4 mg/kg when TOF ratio ≥0.9. Patients were extubated when fully awake and following commands. The primary outcome was definite intraoperative awareness, defined as spontaneous or prompted recall of events between induction and emergence and assessed using the modified Brice questionnaire at 24 hours, 7 days and 30 days postoperatively by blinded investigators. Secondary outcomes included total sevoflurane consumption (MAC-hours), mean end-tidal sevoflurane concentration, recovery times (eye opening, extubation, Aldrete score ≥9), haemodynamic variability (coefficient of variation of HR and MAP), incidence of hypotension/hypertension requiring intervention and PONV within 24 hours. Data were analyzed using SPSS version 28.0 (IBM). Continuous variables are presented as mean ± SD; categorical variables as frequency (%). Student's t-test, Mann-Whitney U test, chi-square, or Fisher's exact test was applied as appropriate. P < 0.05 was considered statistically significant.

## Results:

All 200 randomized patients completed the study and follow-up. Baseline characteristics were comparable between groups (Table 1). No patient in either group reported definite intraoperative awareness at any interview (0% vs 0%, p = 1.000). Two patients in the BIS group and three in the clinical group reported vague dreaming without explicit recall (p = 0.651). BIS monitoring significantly reduced anaesthetic exposure and accelerated recovery (Table 2). Haemodynamic stability was superior in the BIS group (Table 3).

## Discussion:

This randomized trial found no episodes of intraoperative awareness in either BIS-guided or clinically monitored groups, confirming that contemporary balanced anaesthesia with sevoflurane and opioids, when carefully titrated, achieves extremely low awareness rates even without processed EEG monitoring in moderate-risk patients. The most clinically relevant finding was the 24% reduction in sevoflurane consumption with BIS guidance, consistent with multiple previous investigations. This anaesthetic-sparing effect arises from avoidance of unnecessarily deep anaesthesia commonly administered when relying solely on haemodynamic signs, which are influenced by multiple non-hypnotic factors including volume status, β-blockade and surgical stimulation intensity [10, 11]. Faster emergence and shorter PACU stay in the BIS group reflect lower cumulative anaesthetic burden and more precise titration to the minimum effective hypnotic concentration. These benefits improve operating room turnover and reduce nursing workload, potentially offsetting monitor costs in high-volume centres [12]. Superior haemodynamic stability with BIS likely results from proactive rather than reactive anaesthetic adjustment. Clinical signs typically lag behind changes in consciousness, prompting larger corrective swings in volatile agent concentration, whereas BIS enables smooth, anticipatory control [13]. Reduced incidence of both hypotension and hypertension may decrease perioperative cardiac stress, particularly beneficial in patients with coronary disease [14]. The low incidence of awareness in both arms aligns with recent audits showing modern anaesthetic practice has driven rates to 0.1-0.5 per 1000 when muscle relaxants are used judiciously [15]. This suggests BIS may offer limited additional protection against awareness but retains value for drug optimization and recovery. Strength of the study includes strict protocol adherence, triple-time-point awareness assessment up to 30 days, blinded postoperative evaluation and use of sugammadex for rapid reliable reversal. Limitations include inability to blind the attending anaesthetist, exclusion of high-risk patients (cardiac, trauma, caesarean) and relatively short mean surgical duration.

## Conclusion:

In a moderate-risk elective surgical population, BIS monitoring did not reduce the already negligible incidence of intraoperative awareness compared with careful clinical monitoring. However, BIS guidance substantially decreased volatile anaesthetic consumption, accelerated emergence and PACU discharge and significantly improved intraoperative haemodynamic stability. These findings support selective rather than routine use of BIS monitoring, with greatest benefit in prolonged procedures, teaching environments, or when precise anaesthetic titration is desired to optimize recovery and cardiovascular homeostasis.

## Figures and Tables

**Table 1:** Patient demographics and surgical characteristics

**Parameter**	**BIS Group (n=100)**	**Clinical Group (n=100)**	**P-value**
Age (years)	46.8±14.2	48.3±13.9	0.514
Gender (M/F)	58/42	55/45	0.788
BMI (kg/m^2^)	26.4±4.1	25.9±4.3	0.428
ASA status (I/II/III)	38/52/10	35/54/11	0.842
Duration of surgery (min)	128±42	132±46	0.576
Duration of anaesthesia (min)	154±48	158±51	0.612
Type of surgery (general/orthopedic/urology/gynecology)	42/28/18/12	44/26/19/11	0.974

**Table 2:** Anaesthetic consumption and recovery profile

**Parameter**	**BIS Group (n=100)**	**Clinical Group (n=100)**	**P-value**
MAC-hours (sevoflurane)	1.7±0.4	2.2±0.5	<0.001
Mean end-tidal sevoflurane (%)	1.32±0.28	1.71±0.34	<0.001
Additional propofol boluses (mg)	42±68	58± 74	0.142
Time to eye opening (min)	6.8±2.1	9.4±2.9	<0.001
Time to extubation (min)	7.9±2.3	10.8±3.1	<0.001
PACU discharge time (min)	46.2±11.8	56.9±14.7	<0.001

**Table 3:** Intraoperative haemodynamic parameters and adverse events

**Parameter**	**BIS Group (n=100)**	**Clinical Group (n=100)**	**P-value**
HR coefficient of variation (%)	13.6±4.4	18.9±5.8	<0.001
MAP coefficient of variation (%)	11.4±3.9	16.8±5.6	<0.001
Episodes of hypotension (MAP <60 mmhg)	19 (19%)	36 (36%)	0.008
Episodes of hypertension (MAP >100 mmhg >5 min)	14 (14%)	29 (29%)	0.011
PONV within 24h, n (%)	16 (16%)	22 (22%)	0.297
Explicit recall of intraoperative events	0	0	1
